# Regulation of ROS-Dependent JNK Pathway by 2’-Hydroxycinnamaldehyde Inducing Apoptosis in Human Promyelocytic HL-60 Leukemia Cells

**DOI:** 10.3390/pharmaceutics13111794

**Published:** 2021-10-26

**Authors:** Kyung-Sook Chung, Chae-Bin Yoo, Jeong-Hun Lee, Hwi-Ho Lee, Sang-Eun Park, Hee-Soo Han, Su-Yeon Lee, Byoung-Mok Kwon, Jung-Hye Choi, Kyung-Tae Lee

**Affiliations:** 1 Department of Pharmaceutical Biochemistry, College of Pharmacy, Kyung Hee University, 26 Kyungheedae-ro, Seoul 02447, Korea; adella76@hanmail.net (K.-S.C.); aufewig@hanmail.net (C.-B.Y.); ztztzt08@hanmail.net (J.-H.L.); hhlee4083@naver.com (H.-H.L.); qkrtkddms0930@naver.com (S.-E.P.); heesu3620@daum.net (H.-S.H.); suyeonzz1234@naver.com (S.-Y.L.); 2 Department of Life and Nanopharmaceutical Science, College of Pharmacy, Kyung Hee University, 26 Kyungheedae-ro, Seoul 02447, Korea; jchoi@khu.ac.kr; 3 Department of Biomedical and Pharmaceutical Sciences, College of Pharmarcy, Kyung Hee University, Seoul 02447, Korea; 4 Laboratory of Chemical Biology and Genomics, Korea Research Institute of Bioscience and Biotechnology, Daejeon 34141, Korea; kwonbm@kribb.re.kr; 5 Oriental Pharmaceutical Science, College of Pharmacy, Kyung Hee University, 26 Kyungheedae-ro, Seoul 02447, Korea

**Keywords:** 2′-hydroxycinnamaldehyde, reactive oxygen species (ROS), Bim

## Abstract

The present study demonstrated that 2′-hydroxycinnamaldehyde (2′-HCA) induced apoptosis in human promyelocytic leukemia HL-60 cells through the activation of mitochondrial pathways including (1) translocation of Bim and Bax from the cytosol to mitochondria, (2) downregulation of Bcl-2 protein expression, (3) cytochrome *c* release into the cytosol, (4) loss of mitochondrial membrane potential (Δ*Ψ_m_*), and (5) caspase activation. 2′-HCA also induced the activation of c-Jun N-terminal kinase (JNK) and extracellular signal-regulated kinase1/2 (ERK1/2) in HL-60 cells. The pharmacological and genetic inhibition of JNK effectively prevented 2′-HCA-induced apoptosis and activator protein-1 (AP-1)-DNA binding. In addition, 2′-HCA resulted in the accumulation of reactive oxygen species (ROS) and depletion of intracellular glutathione (GSH) and protein thiols (PSH) in HL-60 cells. NAC treatment abrogated 2′-HCA-induced JNK phosphorylation, AP-1-DNA binding, and Bim mitochondrial translocation, suggesting that oxidative stress may be required for 2′-HCA-induced intrinsic apoptosis. Xenograft mice inoculated with HL-60 leukemia cells demonstrated that the intraperitoneal administration of 2′-HCA inhibited tumor growth by increasing of TUNEL staining, the expression levels of nitrotyrosine and pro-apoptotic proteins, but reducing of PCNA protein expression. Taken together, our findings suggest that 2′-HCA induces apoptosis via the ROS-dependent JNK pathway and could be considered as a potential therapeutic agent for leukemia.

## 1. Introduction

Changes in cellular oxidative stress have emerged as a critical event in cancer. Intracellular reactive oxygen species (ROS), which are produced continuously by the mitochondria, have been suggested to regulate the processes involved in cancer cell cycle arrest, senescence, and apoptosis [1]. This can be achieved by reducing the levels of cellular antioxidants such as glutathione (GSH) and ROS including superoxide anion, nitric oxide, peroxynitrite, and hydrogen peroxide [2]. Elevated levels of ROS activate cellular signaling pathways such as mitogen-activated protein kinase (MAPK), nuclear factor kappa-B (NF-κB), Wnt, and Kelch-like ECH-associated protein 1 (Keap1)-nuclear factor (erythroid-derived 2)-like 2 (Nrf2), which participate in modulating apoptosis [3]. This may be attributed to an increase in mitochondrial oxidative stress that causes cytochrome *c* release into the cytosol, leading to caspase activation. Several anticancer and chemopreventive agents generate ROS, which are associated with the apoptotic cell death of tumor cells [2].

Leukemia is a major hematological malignancy that causes mortality and morbidity in different age groups [4]. It is defined as the abnormal proliferation, clonality, and differentiation of immature hematopoietic cells in the bone marrow [5]. Leukemia can be classified into four common types: acute lymphocytic leukemia (ALL), chronic lymphocytic leukemia (CLL), acute myeloid leukemia (AML), and chronic myelogenous leukemia (CML). Among these types of leukemia, AML is characterized by the aggressive growth of hematopoietic precursor cells that interfere with the production of normal hematopoietic cells in the bone marrow [6]. Although refinement of supportive treatment has improved the outlook of patients with AML in the past 30 years, more than half of young adults and around 90% of older patients still die from AML [5]. The majority of AML cells express varying amounts of the transmembrane surface glycoprotein CD33 (observed in approximately >80% of patients with AML). Therefore, the FDA has granted accelerated approval to gemtuzumab ozogamicin (GO), which is a humanized monoclonal antibody that binds with the IgV domain of CD33 for older patients with relapsed CD33-positive AML [7]. In addition, CPX-351 (a dual drug liposomal encapsulation of cytarabine and daunorubicin), enasidenib (a selective oral inhibitor of the mIDH2 enzyme), and midostaurin (a multi-targeted kinase inhibitor active in AML patients with an FLT3 mutation) were approved by the FDA in 2017. Although the past few years have been an active period for the clinical testing and FDA approval of various molecularly targeted treatments using novel agents for AML, there is limited information on side effects including hepatotoxicity, cardiotoxicity, hematotoxicity, and infection [8].

2′-Hydroxycinnamaldehyde (2′-HCA), which is an active compound isolated from *Cinnamomum cassia*, is a cinnamaldehyde derivative [9]. 2′-HCA is known to have anti-tumor effects in various cancer cells, which include the prevention of cell proliferation and induction of apoptosis [10,11,12]. In addition, 2′-HCA has been reported to exhibit various biological activities, including the suppression of β-catenin signaling and epithelial-mesenchymal transition (EMT) in cancer cells [13]. In addition, 2′-HCA has been found to suppress cancer cell proliferation and tumor growth via the activation of pyruvate kinase M2 [14] and signal transducer and activator of transcription 3 (STAT-3) by regulating ERK1/2 and ROS generation in prostate cancer cells [15]. Nevertheless, the role and molecular mechanism of 2′-HCA in human leukemia need to be clarified. In the present study, we provide detailed insights into the mechanism underlying the anti-proliferative properties of 2′-HCA in AML.

## 2. Materials and Methods

### 2.1. Materials and Cell Culture

**Figure 1:**
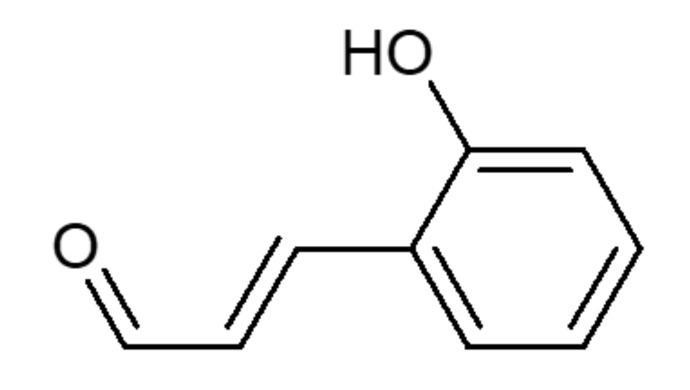
Chemical structure of 2′-HCA.

2′-HCA (Figure 1) was kindly provided by Dr. Byoung-Mok Kwon (Korea Research Institute of Bioscience and Biotechnology) [16]. Primary antibodies against Bax, Bcl-2, caspase-3, PARP-1, α-tubulin, and β-actin were purchased from Santa Cruz Biotechnology Inc (Santa Cruz, CA, USA). Antibody against cytochrome *c* and Annexin V-FITC apoptosis detection kit were purchased from BD Biosciences Pharmingen (San Jose, CA, USA). Antibodies against caspase-9, p-c-Jun, c-Jun, p-ERK1/2, ERK1/2, p-JNK, JNK, p-p38 MAPK, p38 MAPK, and COX-4 were purchased from Cell Signaling Technology (Danvers, MA, USA). z-VAD-fmk, z-DEVD-fmk, and Ac-IETD-CHO were purchased from Calbiochem (Bad Soden, Germany). 3-(4,5-dimethylthiazol-2-yl)-2,5-diphenyl-tetrazolium bromide (MTT), 4′,6-diamidino-2-phenylindole (DAPI), rhodamine 123, propidium iodide (PI), sodium dodecyl sulfate (SDS), dimethyl sulfoxide (DMSO), RNase A, 2′,7′-dichlorofluorescein diacetate (DCFH-DA), and other chemicals were purchased from Sigma Chemical Co (St. Louis, MO, USA). Roswell Park Memorial Institute (RPMI) 1640, Dulbecco’s modified Eagle’s minimum essential medium (DMEM), fetal bovine serum (FBS), penicillin, and streptomycin sulfate were purchased from Life Technologies Inc. (Grand Island, NY, USA).

HL-60 human promyelocytic leukemia, Molt-4 human T lymphoblastic leukemia, U937 human histiocytic lymphoma, K562 human erythroleukemia, HepG2 human hepatoma, SNU-C5 human colorectal adenocarcinoma, A549 human lung adenocarcinoma, and KB human mouth epidermal carcinoma cells were obtained from the Korean Cell Line Bank (KCLB). Cells were cultured in RPMI 1640 DMEM with 10% FBS, penicillin (100 units/mL), and streptomycin sulfate (100 µg/mL). Cells were maintained at 37 °C in a humidified atmosphere of 5% CO_2_.

### 2.2. MTT Assay

Cytotoxicity was measured using the MTT assay. The MTT assay was performed for cytotoxicity measurement using a modified method described [17]. Briefly, the cells (5 × 10^4^) were seeded in each well containing 100 µL of the medium in 96-well plate. After incubation for 24 h, various concentrations of 2′-HCA were added to the 96-well plate. After 48 h, 50 µL of MTT (5 mg/mL stock solution in PBS) was added to each well for 4 h. The medium was discarded and the formazan blue which formed in the cells was dissolved with 100 µL DMSO. The optical density was measured at 540 nm.

### 2.3. Annexin V-FITC/Propidium Iodide (PI) Double Staining Analysis

After treatment with 2′-HCA, apoptotic cells were detected with Annexin V-FITC/PI kit (BD Bioscience, Piscataway, NJ, USA), following the manufacturer’s instruction and analyzed by the fluorescence-activated cell sorting (FACS) cater-plus flow cytometry (Becton Dickinson Co, Heidelberg, Germany).

### 2.4. Detection of DNA Fragmentation

DNA fragmentation was quantitated with DAPI assay as previously reported [18]. In brief, cells were lysed in a solution containing 5 mM Tris-HCl (pH 7.4), 1 mM EDTA, and 0.5% (*w/v*) Triton X-100 for 20 min on ice. After centrifugation at 27,000× *g* for 20 min, the lysate and supernatant were sonicated for 40 s, and the level of DNA was measured by a fluorometric method using DAPI. The amount of the fragmented DNA was calculated as the ratio of the amount of DNA in the supernatant to that in the lysate. The genomic DNA was prepared for gel electrophoresis as previously described [19]. Electrophoresis was performed in a 1.5% (*w/v*) agarose gel in 40 mM Tris-acetate buffer (pH 7.4) at 50 V for 1 h. The fragmented DNA was visualized by staining with ethidium bromide after electrophoresis.

### 2.5. Preparation of Mitochondrial and Cytosolic Fraction and Total Protein

HL-60 cells (2.5 × 10^7^) were collected by centrifugation at 200× *g* for 10 min at 4 °C. The cells were washed twice with ice-cold PBS, followed by centrifugation at 200× *g* for 5 min. The cell pellet was then resuspended in cell lysis buffer (20 mM HEPES-KOH, pH 7.5, 10 mM KCl, 1.5 mM MgCl_2_, 1 mM EDTA, 1 mM EGTA, 1 mM DTT, and 100 µM PMSF) for 30 min on ice. Cells were then homogenized with a glass dounce and a B-type pestle (80 strokes). Cell homogenates were spun at 15,000× *g* for 15 min at 4 °C and the supernatant (cytosolic fraction) was removed while taking care to avoid the pellet. The resulting pellet was resuspended in mitochondrial buffer. For total cell protein extracts, cells were washed with ice-cold PBS and extracted in protein lysis buffer (Intron, Seoul, Korea) for 30 min on ice. Cells were then spun at 15,000× *g* for 30 min at 4 °C and the supernatant (total protein) was used to detection for protein expression.

### 2.6. Western Blot Analysis

For western blot analysis, protein concentration was determined by Bradford assay. Protein samples were mixed with 5 × SDS sample buffer, boiled for 4 min, and then separated by SDS-PAGE gels. After electrophoresis, proteins were transferred to polyvinylidene difluoride membrane. The membranes were incubated with the primary antibody in Tris-buffered saline containing 0.1% Tween-20 (TBS-T) overnight at 4 °C. The primary antibody of the membrane was removed by washing in TBS-T and the membrane was incubated with horseradish peroxidase-conjugated secondary antibodies for 1 h. Following washing in TBS-T, immune blots were visualized by ECL and exposed to X-ray film (GE Healthcare, Midland Park, NJ, USA).

### 2.7. Determination of Mitochondrial Membrane Potential (∆Ψ_m_)

Changes in the Δ*Ψ_m_* were examined by monitoring the cells after double staining with PI and rhodamine 123. After treatment of 40 µM 2′-HCA for 2 h, the cells were incubated with medium containing 5 µg/mL rhodamine 123 for 1 h to determine the mitochondrial membrane potential. The cells were resuspended in 1 mL of minimal essential medium containing 5 µg of PI to assess cell viability. The intensity of fluorescence from PI and rhodamine 123 was measured by flow cytometry. Fluorescence was measured after the cells staining for 30 min at 37 °C.

### 2.8. Detection of ROS Generation

To measure 2′-HCA-induced intracellular ROS level, we used DCFH-DA, which is the most widely used fluorescent probe for the detection of intracellular oxidative stress [20]. The 2′-HCA-treated cells were incubated with 20 µM DCFH-DA for 30 min at 37 °C. The intracellular ROS level was measured by flow cytometry.

### 2.9. Determination of the GSH Level

Cells were washed twice with PBS and treated with 5% trichloroacetic acid (TCA) to extract cellular GSH. The mixture was centrifuged at 13,000× *g* for 1 min to remove the denatured proteins. GSH was determined by the enzymatic method as previously described [21]. To determine the glutathione disulfide (GSSG), the same DTNB recycling assay was performed after using 2-vinylpyridine to remove the reduced GSH [22]. Briefly, 2 μL of 2-vinylpyridine and 6 μL of triethanolamine were simultaneously mixed with 100 μL of sample, followed by incubation in the dark at room temperature for 1 h before initiation of the recycling assay. The kinetics of the reaction was monitored for 10 min. The increment in absorbance at 412 nm was converted to GSH concentration using a standard curve with known amounts of GSH.

### 2.10. Measurement of Intracellular Protein Thiols (PSH)

To measure the intracellular PSH, we performed the assay of intracellular PSH as previously report [22]. Briefly, cells were treated with 5% TCA and then vortexed and kept on ice for 30 min to prepare complete protein precipitation. After centrifugation, the protein precipitate dissolved in 0.1 M Tris-HCl buffer (pH 7.5), containing 5 mM EDTA and 0.5% sodium dodecyl sulfate (SDS). One aliquot of this protein precipitate was reacted with a solution containing 0.1 M sodium phosphate buffer (pH 7.5), 5 μM EDTA, 0.6 mM DTNB, 0.2 mM NADPH, 1 unit/mL glutathione reductase, and another aliquot of the protein solution was treated with 5 mM *N*-ethylmaleimide (NEM) before the reaction to obtain the background value for subtraction. The concentration of intracellular protein thiol was expressed as nmol of SH equivalents/mg protein using GSH as a standard.

### 2.11. Caspase-3 Activity Assay

The caspase-3 activity was measured using a fluorogenic caspase-3 substrate (Ac-DEVD-AFC). Cells were washed once with PBS, resuspended in 400 μL of lysis buffer (20 mM HEPES, pH 7.4, 100 mM NaCl, 0.5% NP-40, and 10 mM DTT) and incubated on ice for 30 min. After centrifugation (12,000× *g* for 5 min), supernatants were collected and immediately measured for protein concentration and caspase activity, or stored at −70 °C until assayed. For the activity assay, 100 μL of cell lysates were placed in a 96-well plate and a caspase substrate was added to each well. Plates were incubated at 37 °C for 1 h and caspase activity was determined from the fluorescence read at 505 nm induced by excitation at 400 nm.

### 2.12. Transfection for RNA Interference

Control small interfering RNA (siRNA) and JNK targeting siRNA (5′-AAAAAGAATGTCCTACCTTCT-3′) specific for humans and mice were obtained from Santa Cruz Biotechnology (Santa Cruz, CA, USA). HL-60 cells (2 × 10^6^) were transfected with siRNA (200 nM) by RNAiFect transfection reagent (QIAGEN, Hilden, Germany) according to recommendations of the manufacturer. After transfection for 48 h, cells were treated with 2′-HCA for 8 h and subjected to analysis.

### 2.13. JNK Kinase Assay

To determine JNK activity, SAPK/JNK kinase assay kit (Cell Signaling Technology, Danvers, MA, USA) was used according to the manufacture’s instruction.

### 2.14. Electrophoretic Mobility Shift Assay (EMSA)

HL-60 cells (5 × 10^6^ cells) were collected by centrifugation at 200× *g* for 10 min at 4 °C. The cells were washed twice with ice-cold PBS, pH 7.2, followed by centrifugation at 200× *g* for 5 min. Nuclear extracts and EMSA assay were performed as described previously [23].

### 2.15. Animals

The male BALB/c nude mice (6-week-old, 20–23 g) were obtained from Nara Biotec Co. (Pyeongtaek, Korea). Mice were inhabited 6/cage/group and were had standard laboratory chow in an animal room with 12 h dark/light cycles at a constant temperature of 20 ± 5 °C.

### 2.16. Xenograft Animal Model

**Figure 2:**
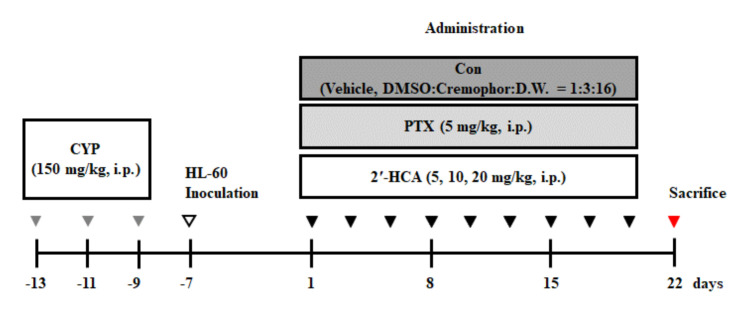
Scheme of the experimental procedure using HL-60 cell-inoculated xenograft animals for assessing the anti-tumor efficacy of 2′-HCA.

The male BALB/c nude mice were subjected to 150 mg/kg cyclophosphamide (CYP) by intraperitoneal (i.p.) injection with three times in 5 days (Figure 2). After CYP injection, HL-60 cells (1 × 10^6^ per site) were inoculated subcutaneously into the right side of the flank of male BALB/c nude mice. Tumor size was checked with a caliper once per 3 days in a week and calculated as V = π/6 × (length) × (width)^2^ [24]. When tumor volume reached around 300 mm^3^, mice were divided into 5 groups and treated with vehicle (DMSO: Cremophor: D.W. = 1:3:16, i.p.), paclitaxel (PTX; positive control, 5 mg/kg, i.p.), and 2′-HCA (5, 10, or 20 mg/kg, i.p.). During the treatment, tumor volume and body weight were measured once per 3 days. On day 21, mice were killed, and tumors were obtained.

### 2.17. Immunohistochemistry (IHC)

Tumor tissues were washed and cleaned by 1 × PBS to fixation with 4% paraformaldehyde. After tumor fixation, the tissues were embedded in paraffin. Tissue section and IHC were performed by Korea Experimental Pathology Inc. (Gyeonggi-do, Korea) and the immune-stained slides were viewed under a light microscope (400×).

### 2.18. Statistical Analysis

All data presented as means ± SD were analyzed by using GraphPad Prism 8.0 Software (San Diego, CA, USA). Student’s *t*-test analysis was applied for statistical analysis to compare all the different groups in the current study. The difference was considered to have statistical significance if *p* < *0.05*.

## 3. Results

### 3.1. 2′-HCA Induces Apoptosis in HL-60 Cells

**Figure 3:**
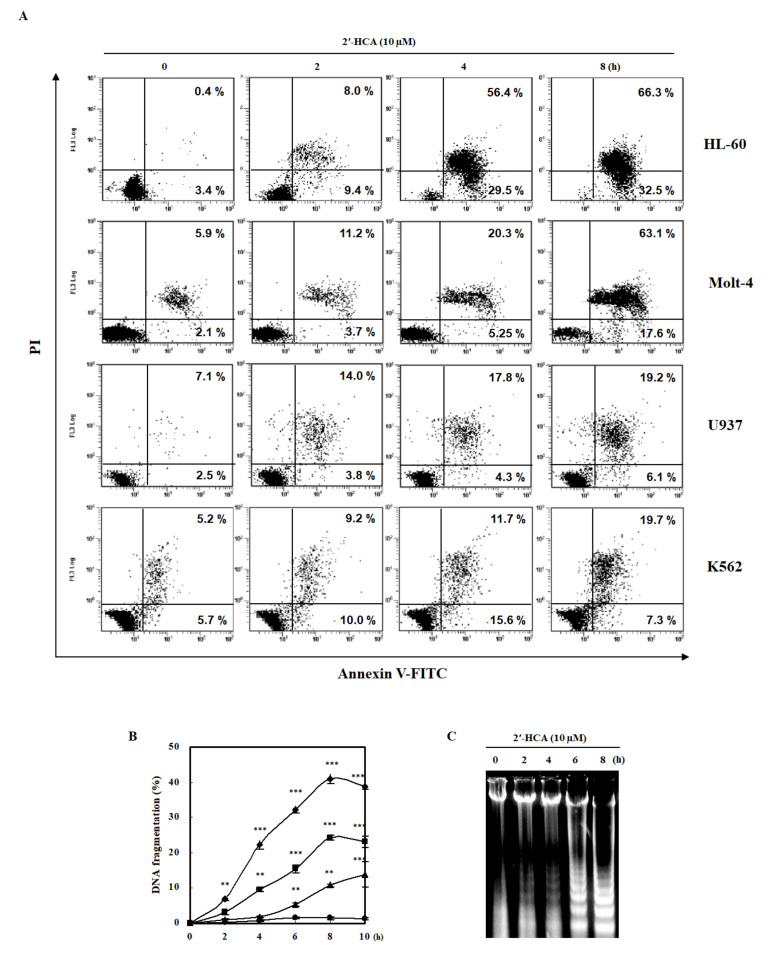
Effects of 2′-HCA on the induction of apoptosis and DNA fragmentation in HL-60 cells. (**A**) HL-60 cells were co-stained with Annexin V-FITC/PI after treatment with 10 µM 2′-HCA to detect the externalization of phosphatidylserine (PS) followed by flow cytometric analysis. HL-60 cells were treated with 10 μM 2′-HCA for the indicated period, and the extent (%) of DNA fragmentation was determined by (**B**) DAPI assay and (**C**) agarose gel electrophoresis as described in the Section 2. Data are presented as the mean ± SD of three independent experiments (●, 0 μM; ▲, 2.5 μM; ■, 5 μM; ◆, 10 μM 2′-HCA). ** p < 0.01 and *** p < 0.001 vs. control group.

Initially, we performed MTT assay to examine the cytotoxic effect of 2′-HCA on various cancer cells (Table 1). Among the tested cancer cell lines, HL-60 and Molt-4 were more susceptible to 2′-HCA. Interestingly, 2′-HCA had no effect on the normal cell viability (IC_50_ > 100 μM, Figure S1) and we speculated that 2′-HCA may be effective for the treatment of leukemia without the cytotoxicity of normal cells. Subsequently, we examined whether the cytotoxicity of 2′-HCA is attributed to apoptotic cell death. As shown in Figure 3A, Annexin V-positive cells were increased in a time-dependent manner after the treatment of the HL-60, Molt-4, U937, and K562 leukemia cells with 2′-HCA. In agreement with the results of MTT assay, 2′-HCA-induced apoptosis was most pronounced in HL-60 cells. Therefore, we selected HL-60 cells to further investigate the apoptotic mechanism. As shown in Figure 3B and 3C, 2′-HCA increased the quantification and laddering pattern of internucleosomal DNA fragmentation in HL-60 cells. These results indicated that 2′-HCA-induced leukemia cell death was caused by apoptosis and that HL-60 cells were highly reactive with 2′-HCA.

### 3.2. 2′-HCA-Induced Apoptosis Is Involved in Mitochondrial Dysfunction and Caspase Activations in HL-60 Cells

**Figure 4:**
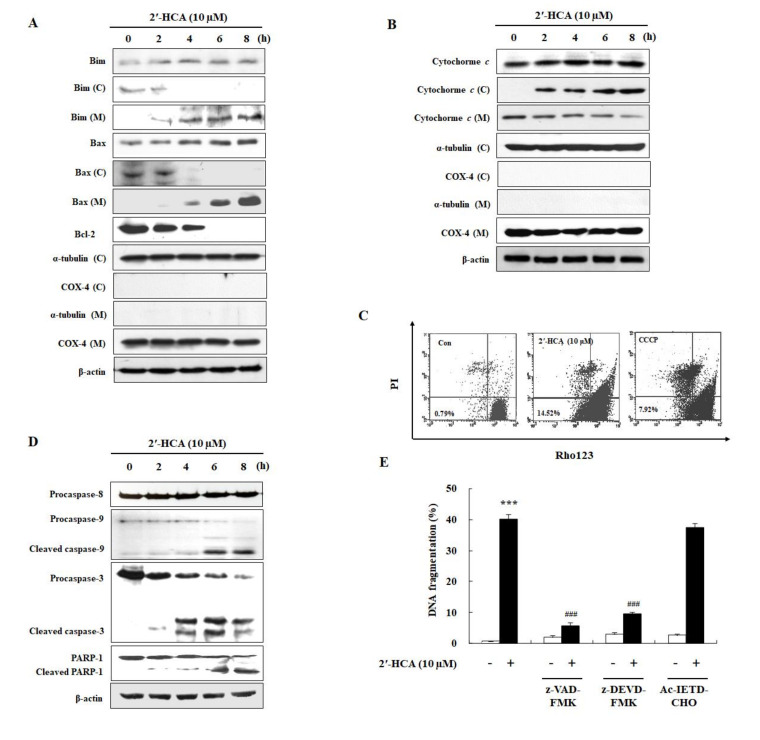
Effects of 2′-HCA on mitochondrial dysfunction and caspase activation in HL-60 cells. (**A**,**B**) Representative western blots showing changes in the protein levels of Bim, Bax, Bcl-2, and cytochrome *c* after treatment with 10 µM 2′-HCA for the indicated times in HL-60 cells (C: cytosol, M: mitochondria). A-tubulin, COX 4, and β-actin were used as internal controls. (**C**) HL-60 cells were treated with 10 μM 2′-HCA for 1 h, and Δ*Ψ_m_* was determined using rhodamine 123 and PI by flow cytometry. (**D**) Representative western blots showing changes in the protein levels of procaspase-9, procaspase-3, cleaved caspase-3, PARP-1, and cleaved PARP-1 after incubation with 10 µM 2′-HCA for the indicated times in HL-60 cells. β-actin was used as an internal control. (**E**) HL-60 cells were pretreated with or without caspase inhibitors (50 μM z-VAD-FMK, 50 μM z-DEVD-FMK, or 50 μM Ac-IETD-CHO) for 1 h and treated with 10 µM 2′-HCA for 8 h. The extent (%) of DNA fragmentation was determined by DAPI assay as described in the Section 2. Data are presented as the mean ± SD of three independent experiments. *** *p* < 0.001 vs. control group, ^###^ *p* < 0.001 vs. 2′-HCA-treated group.

As shown in Figure 4A,B, treatment with 10 μM 2′-HCA time-dependently increased the protein expression and translocation of Bim and Bax from the cytosol to mitochondria and cytochrome *c* release from the mitochondria into the cytosol, and cellular Bcl-2 protein levels were reduced. As cytochrome *c* release is caused by Δ*Ψ_m_* disruption, we next evaluated the effect of 2′-HCA on Δ*Ψ_m_* by flow cytometry after double staining with Rh123 and PI (Figure 4C). The results showed that control cells appeared mostly on the Rh123 high-fluorescence (+) PI (−) (lower right quadrant) field, whereas 2′-HCA-treated HL-60 cells showed an increasing cell population on the Rh123 low-fluorescence (−) PI (−) (lower left quadrant) field. Furthermore, we examined the time-dependent proteolytic cleavage of procaspase-9, procaspase-3, and PARP-1 in HL-60 cells; however, the cleavage of procaspase-8 was not detected (Figure 4D). To determine whether caspase activation is required for 2′-HCA-induced apoptosis, we pretreated HL-60 cells with caspase inhibitors. As shown in Figure 4E, z-VAD-FMK (a broad caspase inhibitor) and z-DEVD-FMK (a caspase-3 inhibitor) inhibited 2′-HCA-induced DNA fragmentation, whereas Ac-IETD-CHO (a caspase-8 inhibitor) did not affect 2′-HCA-induced apoptosis. These observations indicated that the caspase-dependent mitochondrial intrinsic pathway could be involved in 2′-HCA-induced apoptosis.

### 3.3. 2′-HCA-Induced Apoptosis Is Regulated by JNK Activation in HL-60 Cells

**Figure 5:**
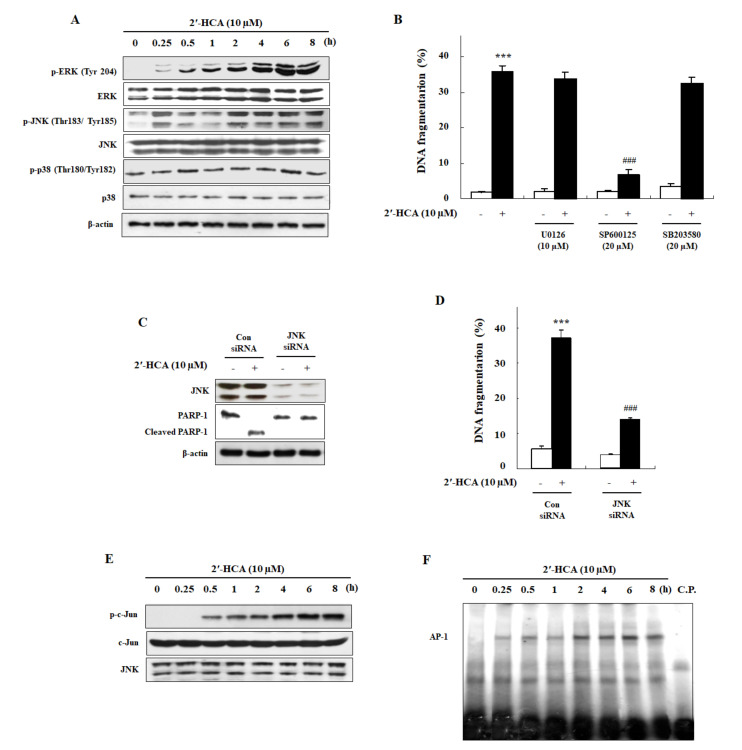
Effect of 2′-HCA on MAPK activation in HL-60 cells. (**A**) Representative western blots showing changes in the protein levels of p-ERK1/2, ERK1/2, p-JNK, JNK, p-p38 MAPK, and p38 MAPK after treatment with 10 µM 2′-HCA for the indicated times in HL-60 cells. β-actin was used as an internal control. (**B**) After pretreatment with MAPK inhibitors (U0126, ERK1/2 inhibitor; SP600125, JNK inhibitor; SB203580, p38 MAPK inhibitor) for 1 h, HL-60 cells were treated with 10 μM 2′-HCA for 8 h, and the extent (%) of DNA fragmentation was determined by DAPI assay. (**C** and **D**) JNK siRNA-transfected HL-60 cells were treated with 10 μM 2′-HCA for 8 h. The levels of protein expression and DNA fragmentation were determined by western blotting and DAPI assay, respectively. (**E**) HL-60 cells were treated with 10 μM 2′-HCA for the indicated times, and JNK activity was determined by JNK kinase assay. (**F**) Nuclear extracts were prepared from 2′-HCA-treated HL-60 cells and analyzed for AP-1 DNA-binding activity by EMSA. A competition experiment using a 5-fold excess of cold oligonucleotides (C.P.) indicated that DNA binding was specific. Data are presented as the mean ± SD of three independent experiments. *** *p* < 0.01 vs. control group, ^###^ *p* < 0.001 vs. 2′-HCA-treated group.

Among the various signaling pathways that respond to stress, the mitogen-activated protein kinase (MAPK) signaling pathway is crucial for apoptosis [25]. To investigate signal transduction events that could contribute to apoptosis, we determined the role of the MAPK pathway in 2′-HCA-treated HL-60 cells. We found that the exposure of HL-60 cells to 2′-HCA resulted in the phosphorylation of JNK, and ERK1/2 but not p38 MAPK (Figure 5A). ERK1/2 phosphorylation was evident as early as 0.25 h after treatment with 2′-HCA and persisted for the duration of the experiment. Moreover, JNK phosphorylation increased at 0.25 h but decreased at 1 h and then again increased thereafter in 2′-HCA-treated HL-60 cells. Commercially available p-JNK inhibitor (SP600125), ERK1/2 inhibitor (U0126), and p38 MAPK inhibitor (SB203580) were used to further determine whether 2′-HCA exerts its sensitizing effects by inhibiting p-JNK, p-ERK1/2, and p-38 MAPK (Figure S2). Interestingly, among the MAPK inhibitors, SP600125 pretreatment significantly inhibited 2′-HCA-induced DNA fragmentation, whereas there was no protective effect by either U0126 (ERK1/2 inhibitor) or SB203580 (p38 MAPK inhibitor) pretreatment up to 8 h (Figure 5B). To rule out the possibility of the nonspecific effect of 2′-HCA on JNK, we analyzed the effect of JNK silencing. As shown in Figure 5C and 5D, the knockdown of JNK by siRNA markedly blocked 2′-HCA-induced PARP-1 cleavage and DNA fragmentation in HL-60 cells, which were similar to the effects observed following treatment with the chemical inhibitor SP600125. JNK modulates the apoptotic pathway including the activation of specific transcription factors, such as an AP-1. [26]. We evaluated JNK activity and AP-1 DNA-binding activity with JNK kinase assay and EMSA, respectively, in 2′-HCA-treated HL-60 cells. As shown in Figure 5E,F, JNK and AP-1 DNA-binding activities were significantly increased by treatment with 10 μM 2′-HCA within 2 h, which was earlier than the onset of apoptosis as detected by caspase-3 activation and apoptotic DNA fragmentation. These observations indicated that the JNK pathway could play a crucial role in 2-HCA-induced apoptosis via the regulation of transcription factors.

### 3.4. Oxidative Stress Is Required for 2′-HCA-Induced Apoptosis in HL-60 Cells

**Figure 6:**
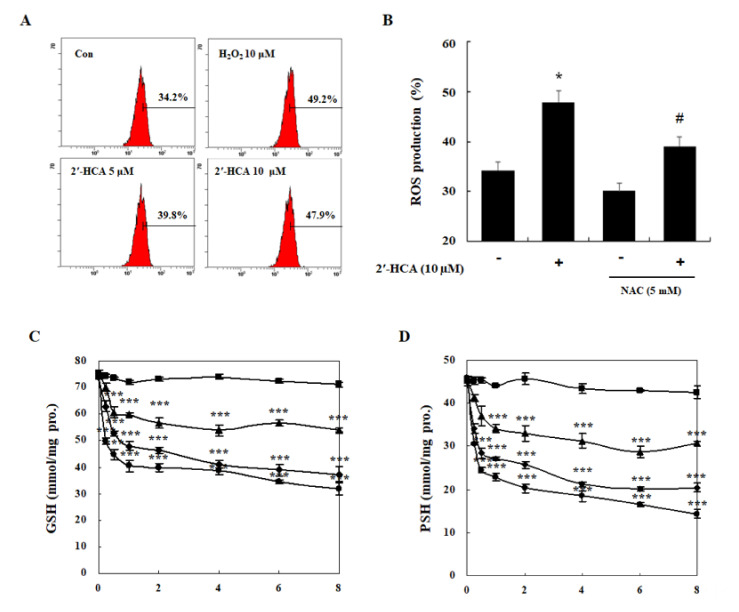

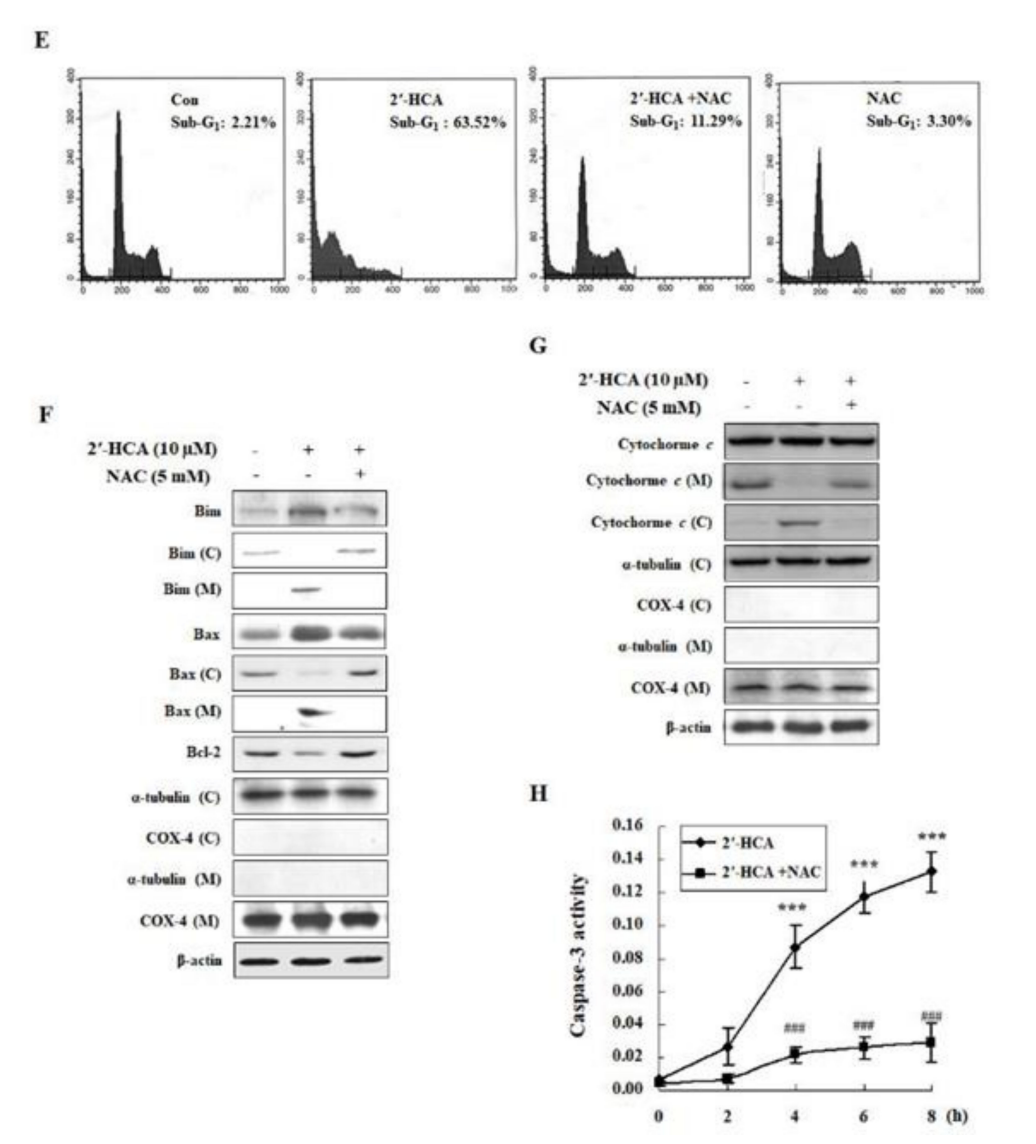
Effects of 2′-HCA on intracellular ROS production in HL-60 cells. (**A**) HL-60 cells were treated with 2′-HCA or H_2_O_2_ for 5 min. ROS levels were determined with a DCFH-DA staining and the fluorescent intensity was measured by flow cytometry. (**B**) After pretreatment with 5 mM NAC for 1 h, HL-60 cells were treated with 10 μM 2′-HCA for 5 min. The levels of intracellular (**C**) GSH and (**D**) PSH were determined in 2′-HCA-treated HL-60 cells (●, 0 μM; ▲, 2.5 μM; ■, 5 μM; ◆, 10 μM 2′-HCA). After pretreatment with 5 mM NAC for 1 h, HL-60 cells were treated with 10 μM 2′-HCA for 8 h, and (**E**) sub-G_1_ and (**F**,**G**) mitochondria-related protein expression was detected by PI staining and western blotting, respectively. α-tubulin, COX-4, and β-actin were used as internal controls. (**H**) The effect of NAC on caspase-3 activity was examined in 2′-HCA-treated HL-60 cells. Data are presented as the mean ± SD of three independent experiments. * *p* < 0.05, *** *p* < 0.001 vs. control group, ^#^ *p* < 0.05, and ^###^ *p* < 0.001 vs. 2′-HCA-treated group.

To confirm whether ROS are involved in 2′-HCA-mediated apoptosis in leukemia cells, we measured the levels of cellular ROS using DCFH-DA with a fluorescence microscope. As shown in Figure 6A, marked ROS generation was observed in 5min after treatment with 10 μM 2′-HCA, and the 2′-HCA-induced ROS generation was significantly reduced with antioxidant *N*-acetylcysteine (NAC) pretreatment (Figure 6B). As increasing evidence has suggested that the intracellular thiol redox status is one of the key mediators of apoptosis in many cell systems [27], we examined whether 2′-HCA-induced apoptosis involves the depletion of intracellular thiols. 2′-HCA rapidly reduced the levels of intracellular GSH and PSH in a time- and concentration-dependent manners and a statistically significant difference was detected as early as 15 min after treatment with various concentrations (2.5, 5, or 10 μM) of 2′-HCA (Figure 6C,D). To examine whether the generation of ROS is a crucial step in 2′-HCA-induced apoptosis, we investigated the effect of NAC on 2′-HCA-induced apoptosis. Pretreatment with NAC decreased the 2′-HCA-induced sub-G_1_ cell population (Figure 6E) and abrogated the protein expression and translocation of Bim and Bax from the cytosol to mitochondria, reduction of Bcl-2 protein expression (Figure 6F), and release of cytochrome *c* into the cytosol (Figure 6G). In addition, pretreatment with NAC significantly attenuated 2′-HCA-induced caspase-3 activity in HL-60 cells (Figure 6H). These results demonstrated that oxidative stress could play an important role in 2′-HCA-induced apoptosis in HL-60 cells.

### 3.5. Reduced Oxidative Stress Attenuates the 2′-HCA-Induced JNK Pathway and Mitochondrial Translocation of Bim in HL-60 Cells

**Figure 7:**
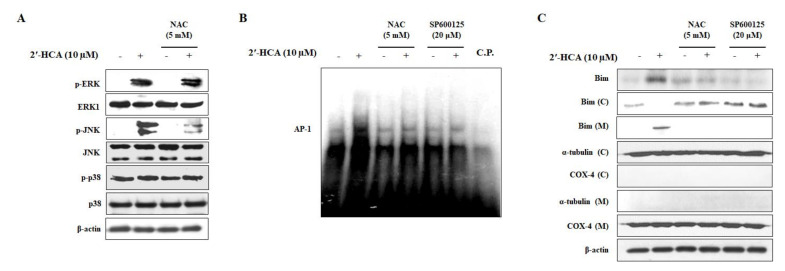
Effect of 2′-HCA-activated ROS on MAPK activation in HL-60 cells. (**A**) Representative western blots showing changes in the protein levels of p-ERK1/2, p-JNK, p-p38 MAPK, ERK1/2, JNK, and p38 MAPK after pretreatment with 5 mM NAC for 1 h followed by treatment with 10 μM 2′-HCA for 8 h. β-actin was used as an internal control. (**B**,**C**) After pretreatment with 5 mM NAC or 20 μM SP600125 for 1 h, HL-60 cells were treated with 10 μM 2′-HCA for 8 h, and AP-1 DNA-binding activity and protein expression and translocation of Bim were analyzed by EMSA and western blotting, respectively. A competition experiment using a 5-fold excess of cold oligonucleotides (C.P.) indicated that DNA binding was specific. α-tubulin, COX 4, and β-actin were used as internal controls.

ROS can promote the activation of JNK, which modulates the activity of the proapoptotic BH3 subgroup of Bcl-2 family proteins such as Bim [1]. As 2′-HCA activated the JNK pathway and AP-1 transcription factor activity, we investigated whether the JNK pathway is associated with oxidative stress in 2′-HCA-treated HL-60 cells. As shown in Figure 7A, pretreatment with NAC effectively attenuated 2′-HCA-induced JNK phosphorylation, but did not affect the phosphorylation of ERK1/2 and p38 MAPK. In addition, pretreatment with NAC completely inhibited 2′-HCA-induced AP-1 DNA-binding activity, indicating oxidative stress-regulated JNK/AP-1 signaling in HL-60 cells (Figure 7B). Furthermore, our results revealed that the mitochondrial translocation of Bim was blocked by treatment with NAC and SP600125, indicating the important role of Bim in the 2′-HCA-activated ROS/JNK pathway (Figure 7C). These results demonstrated that 2′-HCA-induced apoptosis could mediate mitochondrial dysfunction through mitochondrial Bim translocation regulated by the ROS/JNK pathway in HL-60 cells.

### 3.6. 2′-HCA Suppresses Tumor Growth in a HL-60 Xenograft Mouse Model

**Figure 8:**
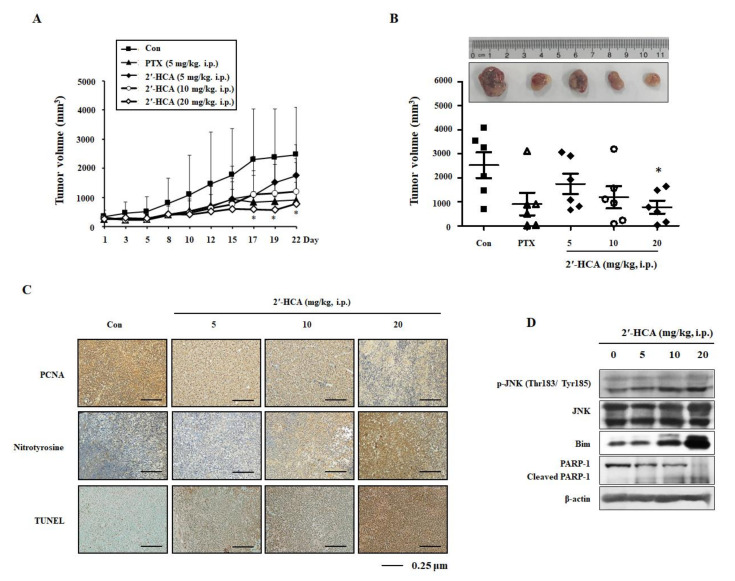
Effect of 2′-HCA on tumor growth in a HL-60 xenograft mouse model. (**A**) The tumor volume (mm^3^) was measured throughout the experimental period, and (**B**) representative tumors were selected after the mice were sacrificed. Data are presented as the mean ± SD (n = 6). * *p* < 0.05 vs. control group. (**C**) The expression levels of PCNA and nitrotyrosine were analyzed by IHC and apoptosis induction was examined by TUNEL assay in tumor tissues. (**D**) Intratumoral expression of p-JNK, Bim, and PARP-1 were evaluated by western blotting. β-actin was used as an internal control.

To evaluate the anti-tumor effect of 2′-HCA, we made a subcutaneous xenograft model of HL-60 cells in immunodeficient mice [28]. As shown in Figure 8A, the average tumor volume was similar in each group at the start of the experiment. After intraperitoneal administration of 2′-HCA, tumor growth was slower than that of the vehicle-treated control group, and the tumor volume was markedly decreased from day 10. In addition, on the last day of the experiment, the tumor volume was significantly smaller in the 2′-HCA administration group (20 mg/kg, i.p.) than in the control group (2525.77 ± 1316.10 mm^3^ vs. 780.47 ± 665.28 mm^3^, *p* < 0.001). Consistently, IHC results showed the marked reduction of the proliferating cell nuclear antigen (PCNA) marker of proliferating cells in the tumor tissues of 2′-HCA-treated mice (Figure 8C). Similar to in vitro results, 2′-HCA treatment increased the expression levels of nitrotyrosine, a marker of ROS, and enhanced apoptosis induction, as demonstrated by TUNEL staining of tumor tissues. Furthermore, western blotting revealed that treatment with 2′-HCA increased the levels of p-JNK, and the pro-apoptotic mediator Bim, resulting in the cleavage of PARP-1 (Figure 8D). During the experimental period, 2′-HCA administration did not affect the body weight, and 2′-HCA showed no toxicity in the HL-60 xenograft mouse model (Figure S3). Taken together, our findings indicated that 2′-HCA could inhibit tumor growth in vivo via ROS generation and JNK activation, which was consistent with the in vitro finding showing apoptosis induction via the ROS-dependent JNK pathway.

## 4. Discussion

ROS play a vital role in various cellular processes under physiological and pathological conditions. Excessive cellular levels of ROS can trigger oxidative stress, which is defined as a severe redox imbalance between the generation of ROS and antioxidant defenses, causing oxidative damage [29]. A moderate increase in ROS can promote cell differentiation and proliferation; however, excessive ROS accumulation causes oxidative stress and damage to cells [30]. Therefore, the anti-tumorigenic signaling of ROS may be targeted in cancer therapy by increasing the production of ROS to toxic levels and exhausting the antioxidant system [31]. Indeed, several studies have reported promising compounds that could elicit ROS generation, leading to apoptosis [32,33]. In our previous studies, we found that costunolide induced apoptosis in human ovarian cancer and leukemia cells via ROS generation and JNK activation [22,34,35], and cinnamaldehyde induced apoptosis via ROS-mediated mitochondrial permeability transition in HL-60 cells [36]. 2′-HCA, which is a derivative of cinnamaldehyde, also induced ROS-mediated apoptosis with STAT-3 inactivation, which was abrogated by GSH or NAC treatment in DU145 cells [15]. Although 2′-HCA can inhibit the growth of human erythroleukemia or skin cancer cells by directly targeting Pim-1 kinase [37], limited studies have been conducted on the ROS-related molecular mechanism of 2′-HCA in human leukemia cells. Therefore, in the present study, we investigated the ROS-mediated anticancer mechanism of 2′-HCA in HL-60 cells. Interestingly, compared with our previous findings, the results here indicated that 2′-HCA (IC_50_ = 4.17 μM) was more cytotoxic than cinnamaldehyde (IC_50_ = 30.7 μM) in HL-60 cells. It is known that the aldehyde group of the side chain and free hydroxy-substituted groups play a critical role in the anti-tumor activity of cinnamaldehydes [38]. Therefore, we believe that the increased cytotoxicity of 2′-HCA may be attributed to a difference in the structure of the H group at the 2′-site of cinnamaldehyde, which is displaced by the hydroxyl group. 2′-HCA induced intracellular ROS generation in HL-60 cells, as detected by the ROS-sensitive fluorescent dye DCFH-DA, which is used as a probe for the specific detection of intracellular hydrogen peroxide rather than superoxide radicals. Similar to our in vitro results, 2′-HCA increased the expression of nitrotyrosine, which can be targeted to elevate cellular ROS levels in tumor tissues. In aerobic organisms, oxidoreductases reactions are always associated with chances of formation of superoxide, the primary ROS molecule in biological systems [39]. Among the oxidoreductases, NADPH oxidase, which is a professional ROS producer, mainly modulates multiple redox-sensitive intracellular signaling pathways by generating ROS molecules via a cross-talk with mitochondria [40]. Mitochondria have generally been considered as a principal source, although estimates range from 2–5% to 0.15% of total O_2_ consumption [41]. Superoxide generation occurs by single electron leakage to O_2_ at complex I, complex III, and possibly the electron transfer flavoprotein-quinone oxidoreductase complex for fatty acid oxidation [42]. Caspase-independent deaths with the mitochondrial release of AIF and endonuclease G are usually associated with complex I-dependent superoxide production, while cytochrome *c* is related to ROS source from complex III because cytochrome *c* is the electron acceptor by complex III, and also has function as a scavenger of superoxide released into the intermembrane space. In the present study, pretreatment with NAC abrogated mitochondria-dependent apoptosis including the translocation of Bim and Bax from the cytosol to mitochondria and the release of cytochrome *c* into the cytosol in 2′-HCA-treated HL-60 cells. Based on our data, we speculated that 2′-HCA-induced ROS can be produced from the mitochondria but it is worth further evaluating the ROS resource involving NADPH oxidase.

ROS have been implicated in the activation of various cellular signaling pathways including MAPK, which can activate cell survival and/or cell death processes such as apoptosis [43]. ROS-mediated JNK activation involved ASK1-dependent/-independent signaling pathways [9]. ASK-1, a member of the MAPK kinase kinase (MAPKKK or MAP3K) family, is part of the MAPK cascade and binds to reduced thioredoxin in non-stressed cells. Under oxidative stress, thioredoxin is oxidized and dissociates from ASK-1, leading to the activation of MAPK pathways, which can promote apoptosis [44,45]. Furthermore, several studies reported that ROS-mediated ASK-1 activation is negatively regulated by Pim-1 and STAT3 [46,47]. Although the phosphorylation of ASK-1 first appeared at 0.25 h and peaked at 2 h but then decreased in 2′-HCA-treated HL-60 cells (Figure S4), JNK phosphorylation presented a biphasic pattern (increased at 0.25 h but decreased at 1 h and then again increased). Based on these data, we speculated that 2′-HCA-induced the pulsatile of JNK phosphorylation can be related to ASK1-independent pathways. Many other ASK1-independent pathways have been identified, including (i) the Src–Gab1 pathway, (ii) the GSTπ pathway, and (iii) the RIP–TRAF2 and membrane lipid raft pathway [48]. Among these pathways, interestingly, either genetic disruption of Src or inhibition of Src kinase activity leads to specific suppression of JNK activation, but not of ERK and p38, in cells treated with H_2_O_2_ [49]. In these regards, we considered that additional confirmative studies are warranted to evaluate the effects of 2’-HCA on ROS-mediated ASK1-dependent or -independent signaling pathway.

Although 2′-HCA also increased the phosphorylation of ERK1/2, the SP600125 JNK inhibitor but not the U0126 ERK1/2 inhibitor significantly reduced 2′-HCA-induced DNA fragmentation in HL-60 cells. Several studies reported the correlations of ERK signaling and stimulating the process of cell deaths [50,51,52], but ERK is crucial for cell proliferation and survival response that counteracts with cell death [53]. Accordingly, we suggest that it is necessary to further investigate whether 2′-HCA-induced ERK activation is related to cell survival. Furthermore, pretreatment with NAC attenuated the 2′-HCA-activated DNA-binding activity of AP-1, which is downstream of JNK. We found that ROS could function as an up-regulator of the JNK pathway in 2′-HCA-treated HL-60 cells. Members of the Jun family of transcription factors (c-Jun, JunB, and JunD) form homodimers or heterodimers among them or with Fos family members (c-Fos, FosB, Fra1, and Fra2), to make up AP-1 protein complexes, which is often portrayed as a nuclear decision-maker that determines cell survival or death in response to cellular stimuli [26]. Some studies have focused on its role in extrinsic apoptosis via JNK, c-Jun/AP-1, and Fas ligand (FasL) in lymphocytes [54,55]. AP-1 transcriptional activity is required for the mRNA expression of Bim, which is a crucial apoptosis regulator that induces Bax activation by inhibiting anti-apoptotic proteins such as Bcl-2 and Mcl-1; this results in increased mitochondrial permeability and apoptosis [56]. In the present study, pretreatment with a JNK inhibitor suppressed not only the DNA-binding activity of AP-1 but also the mitochondrial translocation of Bim in 2′-HCA-treated HL-60 cells. Our in vitro results were further confirmed in a xenograft animal model, which showed an increased expression level of Bim in 2′-HCA-treated tumor tissues. With these results, we can suggest that AP-1-binding site could be bound by c-Jun and activated by 2′-HCA treatment, leading to upregulate the mRNA expression of Bim, which induce the loss of mitochondrial membrane integrity and resulting in apoptosis. Although the correlation between in vitro and in vivo observations supports this mechanistic explanation, it is necessary to further investigate whether the protein binding to the *Bim* AP-1 site is a c-Jun homodimer in 2′-HCA regulated ROS-dependent JNK Pathway.

In addition to the AP-1-responsive gene expression of the JNK pathway, it has been reported that ROS-mediated JNK activation can regulate the phosphorylation and downregulation of anti-apoptotic Bcl-2 proteins [57]. JNK alters the composition of the Bax/Bcl-2 complex by increasing the expression of Bax, leading to the formation of Bax homodimers and resulting in the opening of the mitochondrial permeability transition pore [58]. Consistent with these findings, our results indicated that 2′-HCA induced a time-dependent increase in conformationally changed Bax (Bax/6A7) based on immunoprecipitation with an antibody directed against the NH_2_-terminal region of Bax (Figure S5), suggesting that JNK activation may play a crucial role in the 2′-HCA-induced intrinsic apoptosis pathway in HL-60 cells.

## 5. Conclusions

**Figure 9:**
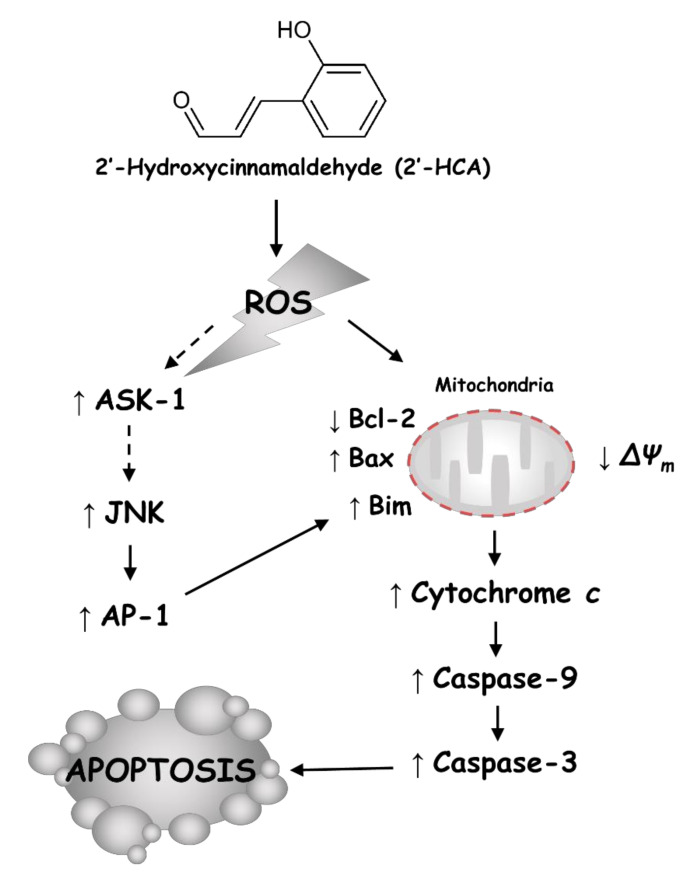
Graphic abstract. The molecular mechanism of 2′-HCA via ROS/JNK/apoptosis in human leukemia.

In conclusion, the current study demonstrated that 2′-HCA could induce apoptosis in human promyelocytic leukemia HL-60 cells via the ROS-dependent JNK pathway involving AP-1-DNA binding, which in turn may increase Bim protein expression, leading to mitochondrial translocation and the subsequent disruption of Δ*Ψ_m_* (Figure 9). Our findings suggest that 2′-HCA could be a useful pharmacologic tool for improving our understanding of basic cellular functions and that 2′-HCA may be considered as a potential therapeutic agent for human leukemia.

## Figures and Tables

**Table 1:** Effect of 2′-HCA on cancer cell growth in vitro.

Cell Line	Origin	IC_50_ (μM) ^(a)^	
		2′-HCA	Cisplatin
HL-60	Human promyelocytic leukemia	4.17 ± 0.62	17.7 ± 2.54
Molt-4	Human T lymphoblastic leukemia	9.14 ± 0.88	9.34 ± 1.12
U937	Human histiocytic lymphoma	23.25 ± 1.98	22.0 ± 2.18
K562	Human erythroleukemia	23.15 ± 2.34	>100
HepG2	Human hepatoblastoma	17.08 ± 1.76	54.6 ± 4.88
SNU-C5	Human colorectal adenocarcinoma	22.77 ± 2.92	27.4 ± 2.76
A549	Human lung adenocarcinoma	33.14 ± 3.76	47.62 ± 3.87
KB	Human mouth epidermal carcinoma	53.04 ± 4.91	56.43 ± 4.99

^(a)^ IC_50_ is defined as the concentration that results in a 50% decrease in the number of cells compared with that of the control cultures in the absence of 2′-HCA. The values represent the mean of three independent experiments.

## Data Availability

Not applicable.

## Supplementary Materials

The following are available online at https://www.mdpi.com/article/10.3390/pharmaceutics13111794/s1, Figure S1: The cytotoxic effect of 2’-HCA on the normal cells. Cells were treated with various concentration of 2’-HCA for 48 h. The cell viability was determined by MTT assay, Figure S2: Inhibition of 2′-HCA-induced MAPK phosphorylation in MAPK inhibitors-treated HL-60 cells. After pretreatment with MAPK inhibitors (U0126, ERK1/2 inhibitor; SP600125, JNK inhibitor; SB203580, p38 MAPK inhibitor) for 1 h, HL-60 cells were treated with 10 μM 2′-HCA for 8 h. Representative western blots showing changes in the phosphorylation and expression levels of MAPK and β-actin were used as internal controls, Figure S3: Effect of 2′-HCA on body weight in a HL-60 xenograft mouse model. Data are presented as the mean ± SD (n = 6), Figure S4: Effect of 2′-HCA on ASK activation in HL-60 cells. Representative western blots showing changes in the protein levels of p-ASK and ASK after treatment with 10 µM 2′-HCA for the indicated times in HL-60 cells. β-actin was used as an internal control, Figure S5: Effect of 2′-HCA on the conformational change of Bax in HL-60 cells. The cells were treated with 2′-HCA (10 µM) for the indicated times. For immunoprecipitation (IP), cells were lysed in EBC buffer (50 mM Tris, pH 8.0, 120 mM NaCl, 0.5% NP-40, 5 µg/ml leupeptin, 10 µg/ml aprotinin, 50 µg/ml PMSF, 0.2 mM sodium ortho-vanadate, 100 mM NaF) for 30 min at 4 ºC. After centrifugation (10,000 g, 5 min), 100 µg of protein was precipitated with Bax antibody for 12 h at 4 ºC, followed by incubation with 20 µl of protein A-Sepharose beads for 4 h. The mixture was washed 4 times with EBC buffer and denatured by boiling in 2 × sample buffer (125 mM Tris–HCl, pH 6.8, 4% SDS, 10% β-mercaptoethanol, 2% glycerol and 0.02% bromophenolblue) for 5 min. The reaction mixture was then resolved by a 12% SDS- polyacrylamide gels, transferred to nitrocellulose membrane and probed with anti- Bax/6A7 antibody. Immunoblotting (IB)was visualized by ECL kit (GE Healthcare, Midland Park, NJ, USA).
