# Supplementary material for: Regulation of ROS-Dependent JNK Pathway by 2’-Hydroxycinnamaldehyde Inducing Apoptosis in Human Promyelocytic HL-60 Leukemia Cells
Source: Pharmaceutics. 2021 Oct 26;13(11):1794. doi: 10.3390/pharmaceutics13111794 (PMC8618870; doi:10.3390/pharmaceutics13111794)
Supplement: Supplementary file 1 [file pharmaceutics-13-01794-s001.zip › pharmaceutics-1393268-supplementary.pdf]

pharmaceutics

Supplementary Materials: Regulation of ROS-Dependent JNK Pathway by 2′-Hydroxycinnamaldehyde Inducing Apoptosis in Human Promyelocytic HL-60 Leukemia Cells

Kyung-Sook Chung [1,†], Chae-Bin Yoo [1,†], Jeong-Hun Lee [1,2], Hwi-Ho Lee [1], Sang-Eun Park [1,3], Hee-Soo Han [1,2], Su-Yeon Lee [1,3], Byoung-Mok Kwon [4], Jung-Hye Choi [2,5] and Kyung-Tae Lee [1,2,*]

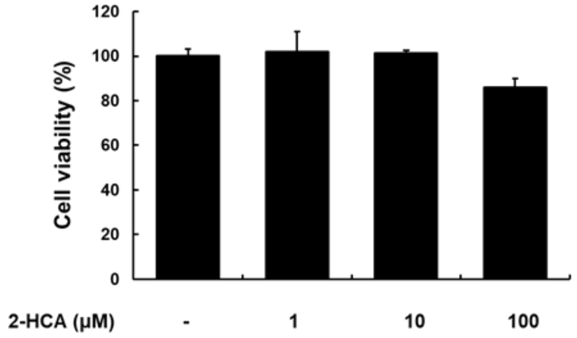

Figure S1. The cytotoxic effect of 2′-HCA on the normal cells. Cells were treated with various concentration of 2′-HCA for 48 h. The cell viability was determined by MTT assay.

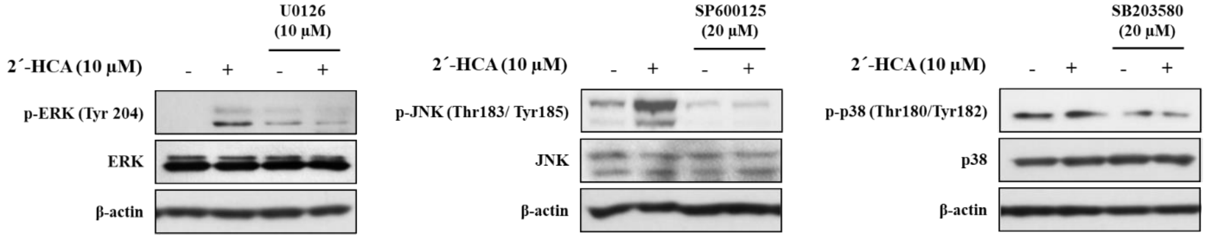

Figure S2. Inhibition of 2′-HCA-induced MAPK phosphorylation in MAPK inhibitors-treated HL-60 cells. After pretreatment with MAPK inhibitors (U0126, ERK1/2 inhibitor; SP600125, JNK inhibitor; SB203580, p38 MAPK inhibitor) for 1 h, HL-60 cells were treated with 10 μM 2′-HCA for 8 h. Representative western blots showing changes in the phosphorylation and expression levels of MAPK and β-actin were used as internal controls.

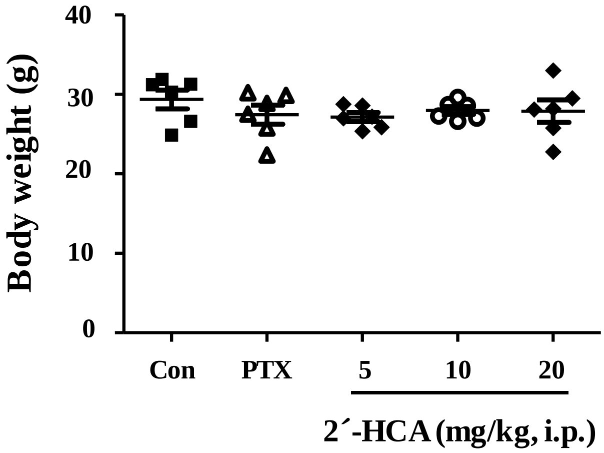

Figure S3. Effect of 2′-HCA on body weight in a HL-60 xenograft mouse model. Data are presented as the mean ± SD (n = 6).

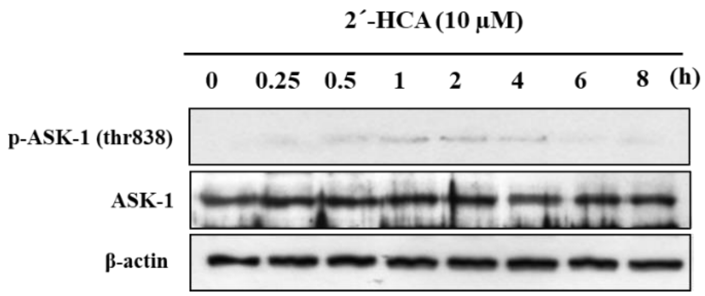

Figure S4. Effect of 2′-HCA on ASK activation in HL-60 cells. Representative western blots showing changes in the protein levels of p-ASK and ASK after treatment with 10 µM 2′-HCA for the indicated times in HL-60 cells. β-actin was used as an internal control.

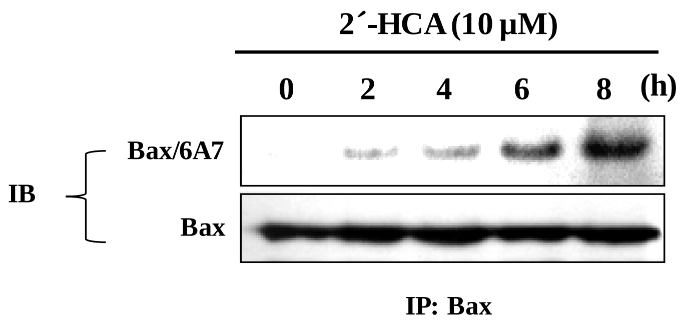

Figure S5. Effect of 2′-HCA on the conformational change of Bax in HL-60 cells. The cells were treated with 2′-HCA (10 µM) for the indicated times. For immunoprecipitation (IP), cells were lysed in EBC buffer (50 mM Tris, pH 8.0, 120 mM NaCl, 0.5% NP-40, 5 µg/ml leupeptin, 10 µg/ml aprotinin, 50 µg/ml PMSF, 0.2 mM sodium ortho-vanadate, 100 mM NaF) for 30 min at 4 ºC. After centrifugation (10,000 g, 5 min), 100 µg of protein was precipitated with Bax antibody for 12 h at 4 ºC, followed by incubation with 20 µl of protein A-Sepharose beads for 4 h. The mixture was washed 4 times with EBC buffer and denatured by boiling in 2 × sample buffer (125 mM Tris–HCl, pH 6.8, 4% SDS, 10% β-mercaptoethanol, 2% glycerol and 0.02% bromophenolblue) for 5 min. The reaction mixture was then resolved by a 12% SDS- polyacrylamide gels, transferred to nitrocellulose membrane and probed with anti- Bax/6A7 antibody. Immunoblotting (IB)was visualized by ECL kit (GE Healthcare, Midland Park, NJ, USA).
